# Towards a conceptualisation and critique of everyday life in HRI

**DOI:** 10.3389/frobt.2023.1212034

**Published:** 2023-09-14

**Authors:** Karolina Zawieska, Glenda Hannibal

**Affiliations:** ^1^ School of Culture and Society, Aarhus University, Aarhus, Denmark; ^2^ Institute of Artificial Intelligence, Ulm University, Ulm, Germany

**Keywords:** human-robot interaction, social robots, everyday life, lived experiences, conceptualisation, critique

## Abstract

This paper focuses on the topic of “everyday life” as it is addressed in Human-Robot Interaction (HRI) research. It starts from the argument that while human daily life with social robots has been increasingly discussed and studied in HRI, the concept of everyday life lacks clarity or systematic analysis, and it plays only a secondary role in supporting the study of the key HRI topics. In order to help conceptualise everyday life as a research theme in HRI in its own right, we provide an overview of the Social Science and Humanities (SSH) perspectives on everyday life and lived experiences, particularly in sociology, and identify the key elements that may serve to further develop and empirically study such a concept in HRI. We propose new angles of analysis that may help better explore unique aspects of human engagement with social robots. We look at the everyday not just as a reality as we know it (i.e., the realm of the “ordinary”) but also as the future that we need to envision and strive to materialise (i.e., the transformation that will take place through the “extraordinary” that comes with social robots). Finally, we argue that HRI research would benefit not only from engaging with a systematic conceptualisation but also critique of the contemporary everyday life with social robots. This is how HRI studies could play an important role in challenging the current ways of understanding of what makes different aspects of the human world “natural” and ultimately help bringing a social change towards what we consider a “good life.”

## 1 Introduction

Whether in reality or only in the realm of expectations, social robots are thought to be increasingly entering our daily life. To achieve a widespread use and acceptance of robots in society, a large part of Human-Robot Interaction (HRI) research seeks to provide scientific and technological solutions for making robotic systems appear and behave socially (Dautenhahn, 2007). Different types of so-called ‘social robots’ have been increasingly envisioned and explicitly promoted as part of our daily life with the goal to assist people with their everyday tasks and activities. The underlying assumption is that the acceptance and use of robotic systems will have a profound impact on our socio-cultural spaces [(Hakli and Seibt, 2017): v], and that robotic technologies will transform “not only how we work but also *how we live*” [(Elliott, 2019): 60] [italics original]. In other words, social robots are considered part of an ongoing fourth industrial evolution that is changing our daily lives (Gonzalez-Aguirre et al., 2021), and the field of social robotics is expected to undergo “explosive growth” [(Dumouchel and Damiano, 2017): 105] in the coming years (whether there is enough empirical evidence to support such claims is a whole different discussion). An important part of such transformation, and the very focus of this article is the impact of robotic systems on our contemporary everyday life.

The goal of this paper is to set the basis for a distinctive research theme and a conceptual framework in the HRI field dedicated to the “HRI of Everyday Life.” We will start by providing an overview of the HRI research dedicated to the subject of everyday life, as well as identify and analyse the key concepts, methods and approaches used in HRI to address different aspects of what is understood as human daily life with social robots. In order to help advance such research, we will follow by proposing to incorporate Social Sciences and Humanities (SSH) perspectives that have long addressed everyday life across different theories and scholar traditions. Given the immense scope of the subject, our goal is to introduce everyday life as a distinctive research theme rather than provide a nuanced disciplinary discussion (it might also be relevant to ask to what extent disciplinary distinctions are possible or even necessary in a situation of increasing interdisciplinarity of SSH research [see, e.g., (Katz and Csordas, 2003; Pedersen, 2016)]). When discussing the key concepts (see Section 4) we rely mainly on the sociological perspectives that are widely established in SSH without prioritising any specific school of thought (e.g., a phenomenological, interactionist, constructivist, or structuralist approach). In other words, our analysis of SSH perspectives is deliberately generic with the goal to leave the doors open for various interdisciplinary explorations that are possible within HRI research and among sociologists themselves.

In SSH research, on the one hand, everyday life has often been perceived as an ordinary or trivial topic (Gardiner, 2000; Sztompka, 2008; Zimmerman et al., 2017); and on the other hand, it has also been seen as a highly complex phenomenon and a human “paramount reality” (Schutz and Luckmann, 1973) that is constitutive of all human thoughts and activities [(Gardiner, 2000): 2]. Not surprisingly, technological transformations have been seen as the key new phenomena in society that constitute an important intellectual challenge for the future sociology and related disciplines [(Tomasi, 2020): 33, after Abbott]. In HRI, a development and implementation of robots into our daily life has been sometimes defined as “the primary incentive” for social robotics [(Weiss and Hannibal, 2018): 399], with the process of wide-spread roboticisation often being perceived as only a matter of time (despite the general public often hesitates to accept social robots for everyday use [(Bartneck et al., 2020): 201]). In other words, in line with technological determinism or optimism, or both, it has often been assumed that as social robotics and AI technologies advance, “Clearly. . . they will likely play an increasingly larger role in our everyday lives and society” [(Bartneck et al., 2020): 203]. Thus, for different reasons but in both the HRI and SSH field, human everyday life is often viewed as a crucial element and the ultimate focus of a respective research agenda. In search for a common denominator for HRI and SSH research, we focus on the concept of ‘lived experience’, that has a potential to build upon the User Experience (UX) approaches that have been widely used in HRI. The key assumption is that social robots and the ways humans engage with such artefacts may constitute an important “extraordinary” element in the otherwise ordinary or taken-for-granted everyday life [(Gardiner, 2000): 6)], and ultimately help transform our social world by unveiling the new possibilities hidden within the everyday.

In particular, in order to help conceptualise the everyday as a distinctive research theme in HRI, we start by providing an overview of how the notion of the everyday has been addressed in HRI research to date, which we identify as related to four different areas (Section 2). We continue by addressing various perspectives the SSH research has to offer on everyday life, particularly in sociology (Section 3), and the elements of everyday lived experienced that we believe will remain largely unaltered in our daily life with social robots (shall it ever materialise) (Section 4). Afterwards, we propose a new conceptual framework that combines both SSH and HRI perspectives and can be used to further explore new aspects of human daily life with social robots, where the everyday is a matter of future and not of today (Section 5). Finally, we discuss potential new developments and roles for HRI research that require engagement with a critical analysis of the contemporary everyday life (Section 6).

## 2 Conceptualisations of the everyday in HRI

While HRI research started from predominantly technical investigations into “understanding, designing, and evaluating robotic systems for use by or with humans” [(Goodrich and Schultz, 2008): 1], its focus has gradually shifted towards more interdisciplinary perspectives with ‘the social’ component at its core (Bartneck et al., 2020). Part of such a shift includes an increasing use of methods and approaches that involve conducting HRI studies “in-the-wild,” i.e., in the expected contexts of use (real-life environments). While still limited, such a trend can be expected to continue: It has been argued that “[t]he fundamental issues for human-robot interaction are in the real world” [ (Kanda and Ishiguro, 2017): 8], and studying robotic systems “in-the-wild” is essential for HRI research (Syrdal et al., 2020) (as discussed below, while HRI researchers refer to the “real world,” sociologists would rather use the term “life-worlds” - an important difference that is more than just a nuance). Further potential developments in HRI research include not only focusing on the social but also becoming more “socially-engaged” (Lee et al., 2022). At the same time, the idea of introducing social robots into people’s daily life often remains more in the realm of motivation for social robotics rather than the actual conceptualisation and empirical study, or is only vaguely addressed, e.g., in terms of the “presence” of robots in our everyday life (Fortunati et al., 2015; de Graaf et al., 2016; Rossi et al., 2020). In other words, as already pointed out elsewhere (Hannibal, 2016; Weiss and Hannibal, 2018), the idea of placing robots in the everyday life contexts lacks a clear understanding of the everyday and is often taken-for-granted in both research and public debates. To the best of our knowledge, in addition to generic calls for an interdisciplinary reflection on the impact of robots on people’s everyday lives (Ray et al., 2008), so far there has been only one attempt made to address everyday life as a distinct research theme in HRI, namely the Everyday-life centred approach (ELCA) (Weiss and Hannibal, 2018). While much more research has been done in this regard within Human-Computer Interaction (HCI) research (Bødker, 2006; Bardzell et al., 2012), the analogies between HRI and HCI are limited. This is because while computer technologies have truly become parts of people’s lives and can be studied as such, social robots are still to a large extent in a research and development phase, and they involve embodied interactions with physical robotic systems (Herath et al., 2020). HCI in turn has been long facing developments towards “ubiquitous computing” where “computation is embedded into the fabric of the world around us. In this world, our primary experience of computation is not with a traditional desktop computer, but rather a range of computationally-enhanced devices—pieces of paper, pens, walls, books, hammers, etc.” [(Dourish, 2004): 19]. Also, although research in HCI and HRI largely overlap in their methods and topics, it has also been recognised that people’s interaction and experience with social robots pose new and distinct challenges (Huang, 2016) which is important to consider when demarcating the everyday life theme in HRI. Therefore, we propose to address the everyday as a distinctive analytical concept and research theme, and to explore its potential to lead to new ways to problematise and engage current HRI research. In order to illustrate why that is the case, the following sections provide an overview of how the everyday has been conceptualised in HRI to date, both explicitly and only in terms of tacit assumptions.

### 2.1 The everyday as settings

In line with thinking of the real world mostly in terms of socio-physical environments, a common way to conceptualise the everyday in HRI research is to refer to *domestic settings* (including in the case of the above-mentioned ELCA (Weiss and Hannibal, 2018)). In general, service robots have often been classified as robots for either professional or personal uses, where the term “personal” has sometimes been used as synonymous with “domestic” (Gonzalez-Aguirre et al., 2021). Social robots are often designed to move around in unstructured and constantly changing environments that are characteristics of people’s homes. The latter are typically viewed as places where private life unfolds. HRI research offers a number of studies conducted in domestic settings where people shared households with robots to a varying degree, whether in real or simulated homes (Forlizzi and DiSalvo, 2006; Koay et al., 2009; Syrdal et al., 2009; Walters et al., 2011; Lee et al., 2022). This is also where one can observe the most frequent use of explicit references to the notion of everyday life, as it is taken-for-granted that daily life takes place at home (see, e.g., (Auger, 2014; de Graaf et al., 2016; Weiss and Hannibal, 2018)). In line with such thinking, social robots have also been studied in *institutional residential settings* where specific groups of people stay and live on a regular basis. For example, people in need of long-term residential care spend much time in nursing homes or rehabilitation centers, where social robots are introduced and used to assist both residents and care givers with daily tasks. Studies that stress the importance of carrying out HRI research in those specific settings (Mannion et al., 2020) are guided by the attention for everyday environments and related robot applications, which can be understood in terms of human daily life with robots. Yet another way of addressing real-life settings in HRI is to conduct HRI studies in *public settings*. This includes, for example, shopping malls (Kanda et al., 2009; Sabelli and Kanda, 2016), airports (Triebel et al., 2016; Joosse and Evers, 2017) or city spaces (Weiss et al., 2008; Weiss et al., 2010) among others, where people engage with robots, and other persons, as part of their daily life. Such an approach opens a discussion into what makes a given task or action “daily,” or what is a relationship and distinction between the private and public domain in people’s everyday life, particularly if we also include virtual spaces. For example, a recent study in digital anthropology has described smartphones as ‘transportal homes’, i.e., new places within which we live (Miller et al., 2021). Also, it has been argued that the actions we pursue in a public and private sphere are equally “everyday” [(Sztompka, 2008): 31]. An alternative approach includes addressing everyday settings in terms of “contexts” for people’s daily experiences (e.g., aging) and related engagement with robotic technologies (Lee et al., 2016). In any case, regardless of their specific features and distinctions we make, it is important to note that addressing everyday settings is often instrumental to investigation of other subjects relevant for HRI research and a daily use of robots, e.g., human trust or acceptance of robots in daily life (Ray et al., 2008; de Graaf et al., 2016; Kuhnert et al., 2017) rather than a research theme in its own right. At the same time, domestic/residential and public settings together make everyday environments that constitute a solid basis for further conceptualisation of everyday life in HRI in terms of a ‘living space’ (see Section 5.1).

### 2.2 The everyday as activity

Another way to conceptualise the everyday in HRI research is in terms of the tasks or activities that social robots are intended to be used for, i.e., specific application domains (Weiss and Hannibal, 2018). While robotic systems used in the contexts of, e.g., healthcare, warfare, manufacturing, rescue, or space exploration are required to carry out very specialised tasks, social robots are typically designed to assist people with general activities in their daily life, e.g., helping people with exercising, cleaning, eating, learning, cooking, socialising, or shopping (Lum et al., 2020; Huang and Huang, 2021). This is how, while supporting everyday tasks, robots may become “everyday objects” (Kaplan, 2005). From this perspective, the everyday refers to those parts of life that occur on a regular basis and that most people are familiar with. A successful application of social robots in this context is also dependent on how well such robots can be integrated into or adapt to people’s habits or routines of actions or behaviour (the goal that can be difficult for technology to achieve, especially with regards to everyday household routines (de Graaf et al., 2016)), and on the degree the interaction with robots can be perceived as ‘natural’. Since this kind of activities is to a large extent considered habitual or mundane, social robots are often required to meet the needs of people through some form of personalisation or basic awareness about which social norms or scripts can be used for the various daily activities (Lee et al., 2012). All in all, a focus on robot applications and related human activities offers a potential for a more holistic conceptualisation of everyday life in HRI, as it covers a variety of domains from domestic assistance, through education or transportation, to healthcare.

### 2.3 The everyday as population

Yet another way to address the everyday in HRI research is to look at the target population intended to use social robots. In general, HRI studies in the real-life settings may involve various types of participants, from randomly selected individuals, through robot users to the actual robot owners (Lee et al., 2022). Those HRI studies that explicitly address the subject of everyday life often refer to people involved in the studies as “lay” or “naïve” users (Takayama et al., 2008; Theofilis et al., 2015; Suguitan and Hoffman, 2019; Rossi et al., 2020). The term “naïve” however is problematic as it does not accurately describe a lack of only a specific type of (technical) knowledge about robotics systems it points to (rather than a quality of being generally naïve), and it undermines people’s active role in shaping technology by their “everyday living with it” [(Bakardjieva, 2005): 38]. An alternative term that we also view as more suitable for HRI research is that of ‘non-expert’ users, or ‘lay experts’ (Weiss and Spiel, 2022) (of course, we are not implying that people are not experts in other areas and everyday activities, including in those that roboticists may have a little understanding of). Such a term is useful not only in referring to people’s limited knowledge or experience regarding robots but also in explicitly situating HRI research in everyday contexts (Lee and Sabanović, 2014; Papagni et al., 2022). It also allows looking at the study participants with the attention for wider socio-cultural and professional backgrounds, e.g., as non-expert caregivers (Louie and Nejat, 2020), rather than only “users” of a given robot (the overall need for a greater contextualisation of HRI studies has been by now well-recognised and articulated (Lee et al., 2016; Lee et al., 2022)). This is particularly true for social robots that in principle are designed for non-expert populations (e.g., elderly, children, or school-teachers) that dominate studies “in-the-wild” (Lee et al., 2022).

### 2.4 The everyday as methodology

Perhaps the most holistic view of the everyday, and a perspective closest to SSH research, is the HRI method of studying robots and human-robot interactions “in-the-wild.” In general, conducting studies in the expected contexts of use, or field studies [6], refer to real-life environments that may vary form simulated real-world settings to the actual socio-physical spaces outside the laboratory. Such a methodology is viewed as instrumental in improving robot design and functionalities [including with the involvement of the study participants as co-designers (Ostrowski et al., 2022)] in a way that robots best fulfil people’s expectations, preferences and needs, and fit into people’s lives. Thus, field studies help increase ecological validity of the HRI studies, i.e., generalisability of the findings to the real world^1^ [6]. At the same time, studies ‘in-the-wild’ involve serious technical and methodological challenges and play only a complementary role in the contemporary HRI research (Jung and Hinds, 2018; Syrdal et al., 2020). The very idea of designing robots for everyday life has sometimes been described as going “on the absolute outer limits of workability” [(Maibaum et al., 2022): 472] in robotics. For the purposes of this work, it is important to emphasise that studies ‘in-the-wild’ involve not only a specific type of environments but also the type of users and research subjects that cannot be successfully addressed in the laboratory settings. Also, such studies allow addressing the underlying socio-cultural assumptions on both the robot developers and end-users’ side [17], or social environments and group dynamics that otherwise remain unnoticed or obscure (Jung and Hinds, 2018; Lee et al., 2022). An important feature of the studies ‘in-the-wild’ is an emphasis on and efforts to conduct long-term studies (Ostrowski et al., 2022) that would hence address not only spatial but also temporal settings typical of our daily life, and the type of activities such spatio-temporal settings involve. For example, the Everyday-life centered approach (ELCA) emphasises the need to capture and record encounters between humans and robots in unstructured situations and real-time. The key three dimensions of everyday life according to ELCA include actions, meaning and materiality (Weiss and Hannibal, 2018). All in all, while the HRI field is yet to assist development of theories, methods, technologies as well as institutional frameworks and practices that would allow studying social robots ‘in-the-wild’ systematically and on a large-scale, a large part of HRI research has been gradually shifting towards the subject of everyday life.

## 3 SSH perspectives on the everyday and HRI research

Across multiple and sometimes very different social and cultural theories of everyday life, there has been a general agreement that the everyday constitutes a dominant element of human existence and social world [10, 19]. In the capacity of the everyday to be “the largely taken-for-granted world” [(Gardiner, 2000): 2] or “the reality that seems self-evident” [(Schutz and Luckmann, 1973): 3], everyday life has often remained clandestine or overlooked in SSH research. It is only recently that the everyday has gained increased (and renewed) interest in SSH and has been recognised as a research topic in its own right and theorised as such (Adler et al., 1987; Gardiner, 2000; Bennett et al., 2004; Sztompka, 2008; Neal and Murji, 2015; Zimmerman et al., 2017). As the society changes, the lines of SSH inquiry into the everyday inevitably evolve, to include, for example, feminist, cultural and postmodernist perspectives (Gardiner, 2000). Incorporating everyday life into HRI research can be seen as developing one of those new perspectives that is potentially promising not only for a successful integration of social robots into society but also advancing interdisciplinary investigations of the contemporary everyday.

In general, different aspects of the everyday has always been addressed in SSH to a varying degree, and everyday life has been a fundamental research problem, particularly in sociology. Depending on the approach, different disciplines and perspectives, e.g., social phenomenology, or micro-sociologies, have explored a variety of human daily practices, knowledge systems and social facts that together constitute human social existence (Gardiner, 2000; Jacobsen, 2008; Sztompka, 2008; Zimmerman et al., 2017). Some approaches such as ethnomethodology have brought a particularly rich contribution to the sociology of everyday life, with different, sometimes competing views, methods and developments in the study of the everyday (Garfinkel, 1967; Attewell, 1974; Atkinson, 1988). Over time, there has been a shift in SSH from addressing everyday life as a largely homogenous, unproblematic and fixed feature of social life produced by specific structural forces towards conceptualising daily life as a highly complex and fluid reality that constitutes a mediator between the individual agent and the social structure, and is subject to change (Gardiner, 2000; Bennett, 2005). The status of everyday life as a research, theoretical and political subject, and its history and recent developments in SSH, is a fascinating topic *per se* and a matter of countless disciplinary discussions (Gardiner, 2000; Jacobsen, 2008; Sztompka, 2008; Olson, 2011; Neal and Murji, 2015; Ludtke and Ludtke, 2018) (just as the affirmation and centrality of everyday life in modernity and postmodernism (Taylor, 1989; Featherstone, 1992; McRobbie, 2003), or the plurality of the present-day sociologies and their perspectives on human social life (Sztompka and Burawoy, 2011)). What different perspectives have in common is that human everyday life has long been understood as the realm of the ordinary, or “the common-sense world” (Schutz and Luckmann, 1973) (the approach sometimes echoed in the HRI arguments for the study of “ordinary people” and their use of robots in daily life (Weiss and Hannibal, 2018)), which can be viewed as both an obstacle and an asset in SSH research. Over time, there has been a growing recognition of the importance of the ordinary or “the obvious” (Zimmerman et al., 2017). This includes the need to face a paradox of how to “give significance to what is insignificant” [(Olson, 2011): 176]), and efforts to “take the ordinary seriously as a category of analysis” [(Neal and Murji, 2015): 811], and to develop critical knowledge of our understanding of the “prosaic” [(Gardiner, 2000): 6]. The very understanding of the ‘ordinary’ in this context has been changing. For example, it has been argued that everyday life equally involves all people, irrespective of their class or other defining characteristics, and hence, it concerns as much elites as common people [(Sztompka, 2008): 31]. Others have argued that everyday life can also be seen as the domain of the “extraordinary,” with all the ambiguity, fluidity, and a transformative capacity it involves (Gardiner, 2000; Neal and Murji, 2015). From this perspective, the everyday can be seen as “a site of normativity,” as much as “a site of resistance” (Neal and Murji, 2015), where individuals and societies have the potential to transform the existing social conditions, to the point of searching for utopia “in the here and now, through the transfiguration of everyday life” [(Gardiner, 2000): 25]. Perhaps in line with such thinking, a large part of studies of the everyday have focused on people, practices and spaces that tend to be marginalised, anonymous or otherwise “unofficial” [(Gardiner, 2000): 8-9] (Jacobsen, 2008; Ludtke and Ludtke, 2018). Such an approach is at least partially due to the need to take “an explicit ethico-political stance” [(Gardiner, 2000): 9] when pursuing a critical study of the everyday, and contextualise the analysis within wider sociohistorical developments [(Gardiner, 2000): 7]. All in all, by the 21st century, the area of everyday life studies has been well-established, to the point of sometimes being considered a distinct field with its own canon and disciplinary developments, and new challenges to address (Sztompka, 2008; Olson, 2011; Neal and Murji, 2015).

## 4 The everyday and lived experience

Not surprisingly, the concept of everyday life goes hand in hand with that of lived experiences. This is because the notion of everyday life points to the existence “as it is lived” [(Gardiner, 2000): 1], and “presupposes a focus on the human being who lives it” [(Bakardjieva, 2005): 37]. This is also one of the reasons why, everyday life is often addressed in sociology in terms of ‘life-worlds’. Such an approach to a large extent originated from the work by Husserl (2002) as he took a critical stand towards scientific inquiry where abstractions from everyday appearances were prioritised. Since life-worlds essentially aim to capture the worlds of experience, such an approach brings the attention to human subjects who experience the world and give meanings to their experiences that in turn constitute the order of reality [(Schutz and Luckmann, 1973): 5, 23] (hence, a frequent use of the term in its plural form). On the one hand, particularly in the philosophical phenomenological tradition, experiences are viewed as highly subjective, where “a lived experience is always essentially *one’s own* direct experience” [(Burch, 1990): 135] [italics original]. Particularly with the work by Heidegger (1967), Sarte (1984), de Beuvoir (1953) and Merleau-Ponty (2012) such perspectives were developed into the existential-phenomenological tradition, which related these direct experiences of the world with investigations and discussions about the (universal) human condition. On the other hand, different sociological perspectives, especially symbolic interactionism (Prus, 1996), emphasise the intersubjective character of the human world, where the world is essentially known and lived in common with others (Schutz and Luckmann, 1973). Just as the everyday “requires the inclusion of almost everything” [(Weiss and Hannibal, 2018): 399], the “lived” character of human existence also concerns literally all its aspects, e.g., lived experience of time, space and the body [(Gardiner, 2000): 75]. Also, many of our everyday lives and experiences lay outside the realm of conscious reflection. As a result, defining “lived experience” and “everyday life” is highly problematic. In Gardiner’s words, “Given the habitualized and recurrent nature of daily life, it is difficult to conceptualize or describe in theoretical terms, mainly because it is profoundly *lived*, and experienced as ceaseless recurrence” [(Gardiner, 2000): 87] [italics original].

For the purposes of this work, we provide an overview of those features of everyday experiences that we consider as being widely agreed upon and particularly useful for HRI research. These are also the features of human existence and elements of the SSH theories of the everyday that we consider as directly suitable for the HRI research, and the parts of human everyday experience that will not change with the expected introduction of social robots into daily life (at least not significantly nor immediately). The underlying assumption is that while the analysis of everyday life focuses on the third-person perspective (when looking at “life-worlds”), investigation of lived experiences always involves first-person accounts. Both approaches suit HRI studies that involve mixed methods research, from behavioural and observational measurements [e.g., (Siegel et al., 2009; Kont and Alimardani, 2020)], to tools designed for subjective evaluations and measures of the robot performance and interaction [e.g., (Siegel et al., 2009; Winkle et al., 2019; Hannibal et al., 2022)].

### 4.1 Being taken-for-granted and habituality

Across different SSH perspectives there is a consensus that the everyday is largely self-evident in people’s lives or taken-for-granted (Schutz and Luckmann, 1973; Gardiner, 2000). This is due to the very content of everyday life that involves common-sense and habitual meanings and activities, i.e., “the routine, taken-for-granted experiences, beliefs and practices; the mundane ordinary world” [(Featherstone, 1992): 160]. A key element of daily life in this context is its recurrent character and a degree of familiarity it implies. This is also why everyday life is generally considered difficult to grasp and life-worlds inherently “intransparent” [(Schutz and Luckmann, 1973): 169]. Examples of sociological perspectives that have explored the taken-for-granted character of everyday life include particularly ethnomethodological research that have also started to appear in some HRI studies (Pitsch and Koch, 2010; Jarske et al., 2020; Yamazaki et al., 2022; Pelikan and Jung, 2023) (its focus on micro-interactions and extensive use of conversation analysis makes ethnomethodology particularly attractive for HRI research that involves human-robot social interactions). To what extent and whether people actually approach their daily world “unreflexively” is subject of different interpretations (to the point of considering the “extraordinary” a common component of the everyday, or everyday reflexivity as consisting of unconscious elements too [(Gardiner, 2000): 6]). On the one hand, a self-evident quality of everyday experiences is limited, since the life-world also includes the provinces that are yet unfamiliar and undetermined for a given person [(Schutz and Luckmann, 1973): 167], and every human experience has potentially unlimited new explications [(Schutz and Luckmann, 1973): 169]. On the other hand, everyday life tends to provide a stable order to people and what often seems to be an unalterable horizon of action [(Gardiner, 2000): 5]. This is true as long as people’s everyday experiences and their meanings remain uncontradicted. The moment disruptions of daily routine occur, when what is familiar becomes defamiliarised, or incongruent with our previous experiences, an opportunity for increased reflexivity, and consequently a social change emerges [(Gardiner, 2000): 19-20; (Schutz and Luckmann, 1973): 11]. In line with such thinking, and from a postmodernist perspective, everyday experience has sometimes been viewed as a site of continuous struggle and contestation, and hence far from being ordinary or unproblematic. In this sense, everyday life constitutes a taken-for-granted reality in plurality of forms and meanings, and everyday experiences are highly diversified and complex (Sandywell, 2004).

Perhaps for different reasons (e.g., to address technical and design challenges), HRI and robotics research too has been concerned with the complexity, “wickedness” (Bischof et al., 2020) or ‘messiness’ of the human real-world (Auger, 2014; Dautenhahn, 2018; Matarić, 2018; Bartneck et al., 2020). This applies particularly to the complexity of the real-world problems and of the related data and socio-physical environments, as well as the unpredictability of human behaviour that often make it difficult to plan and execute HRI studies ‘in-the-wild’. The very use of robots in everyday worlds has sometimes been described as “an enormous challenge” (Bischof et al., 2020) and “an absolute borderline case” (ibid.) for robotics. Also, the core of HRI research involving social robots focuses on the “natural” robot design and human-robot interactions. What is “natural” for humans and for the way they interact with and perceive robots is debatable, but in social robotics and HRI it includes exploiting those human characteristics and behaviours that appear to be largely unconscious or “automatic” (particularly with regards to human sociality and a tendency to anthropomorphise (Zawieska et al., 2012)). Such an approach has the potential to go beyond studying only human-robot interactions and build upon the existing SSH scholarship to systematically theorise and empirically investigate human everyday life as a whole. This may include not only studying what makes social robots and related interactions ‘natural’, but also deliberately exploring the element of the non-human or “extraordinary” (the idea inherent in the design that limits robot human-like features to the minimum (Duffy, 2003; Zaga et al., 2017)), that challenges a familiar or self-evident character of the everyday, particularly the underlying assumptions with regards to what it means to be human.

### 4.2 Immediacy and embodiment

Traditionally, different theories of everyday life have emphasised the immediacy of lived experiences. This conveys the image of persons as embodied subjects who engage with other people and the world around them through bodily experiences. In other words, “[e]veryday life is bodily life” [(Silverstone, 2002): 764]. The latter may refer to body-related features, e.g., age, or more broadly, particular material resources and limitations (Silverstone, 2002), as well as spatial and temporal immediacy, that together contribute to how we experience our everyday life, the Other and the social world. In a wider sense, a characteristic of being embodied and socially embedded points to the contextuality of human experiences or specific phenomena such as the human mind [(Linell and Valsiner, 2009): xxviii]. Immediate experiences are often synonymous with lived experiences and directly opposed to different forms of theoretical and cognitive abstractions. The emphasis on the immediacy of experience implies also the emphasis upon the present, and hence, an overall “non-reflexive sense of immersion” [(Featherstone, 1992): 161] in the everyday. Thus, just everyday life, the actual lived experiences are “pre-reflective,” and hence difficult to grasp in their immediate manifestation [(Finlay, 2002): 533]. Past experiences or any new experiences become part of our current “stock of knowledge” within the life-world that, unless it is contradicted, constitutes a taken-for-granted valid reference schema in the everyday (Schutz and Luckmann, 1973). At the same time, it is important to note that the immediacy of experiences is subject to gradation between the immediate and mediate experiences (Schutz and Luckmann, 1973). In the times of a mediated culture and globally mediatised world, the concept of unmediated everyday life has sometimes been fully rejected (Sandywell, 2004), and mediation described as central to our everyday (Silverstone, 2002). This is particularly true for technology-enabled communication and related meaning-making processes, with the media becoming “the second order paramount reality” [(Silverstone, 2002): 763]. From a postphenomenological perspective, technologies have even lost their mediation role since they are no longer “between” humans and their world, as they merge with the human body and experience (Verbeek, 2012). An alternative approach is to address everyday life as a space or an intersection that overcomes different dualisms and dichotomies of the modern thought, e.g., between mind/body, abstract/concrete, immediate/mediate, or ordinary/extraordinary (Sandywell, 2004). This also applies to lived experience whose immediacy and bodily nature is an important but not the only possible characteristic, particularly under and after postmodernism. From a methodological point of view, it is important to note that despite their immediacy, human experiences can be represented and studied as such, e.g., as textual representations of the lived experience of femininity [(McRobbie, 2003): 153], or visual representations produced by the study participants that represent specific aspects of their human experience (Lenette and Boddy, 2013).

One could argue that the emphasis on immediacy and embodiment goes to the core of HRI research. Robots are typically physically embodied systems, and the way human-robot interactions and human behaviours are understood and studied in HRI involves a significant degree of spatial and temporal immediacy. In other words, while HRI research often recognises the potential of robots to impact our everyday life as a whole, in practice, when studying human daily life with robots, the HRI studies typically address a close proximity and direct interaction with robots. We propose this is a major area for developments and experimentation in HRI, when addressing long-term human engagement with robots outside the laboratory and online settings. This may include an overall gradual shift from studying “interaction,” “interaction experience,” or “user experience” towards developing a more holistic view of “lived experiences” on both an individual and societal level. Such an approach requires attention for the factors that go far beyond a person’s past or present immediate experiences involving robots, or a related novelty effect, and addressing people’s “life-worlds” instead.

### 4.3 Situatedness and tangibility

When discussing lived experiences in SSH in connection to the immediacy and bodily nature of such experiences, the emphasis is also on the situatedness of lived experiences in a concrete space and time. What follows is a focus on “concrete persons” and a “concrete world” (Gardiner, 2000; Heller, 2015) that are always located within specific sociohistorical and material conditions (as against theorising an abstract, disengaged and purely cognitive relation a person may have with the Other and a related lived environment [(Gardiner, 2000): 48-50]). Also, the emphasis on the ‘concrete’ involves focusing on “life-worlds” that refer to the worlds of daily existence and reality of specific persons (Sandywell, 2004), with a particular attention for “action” or “practice” that largely contribute to the content and structure of everyday life (Schutz and Luckmann, 1973; Heller, 2015). This has important methodological implications for SSH research (and potentially HRI), since at least in principle, “if anything, everyday life is certainly ‘visible’, and therefore observable” [(Sztompka, 2008): 24]. At the same time, as already mentioned, routinised or habitual elements of everyday life can be difficult to grasp or articulate due to their quality of being largely outside of the conscious thought (Weiss and Hannibal, 2018). While the term “situatedness” has been well-established in robotics as it points to robotics systems existing in and being affected by complex, dynamic environments (Matarić, 2006) (and in HCI, particularly through Suchman’s early ethnomethodological research on situated actions (Suchman, 1987; Suchman, 2007)), in HRI, a situated character of everyday experiences typically translates into the subject of “contextualisation.” While still limited in number and scope, more and more HRI studies have been addressing different contextual factors that play a role in human actions, attitudes and the related use of robots, from individual features and backgrounds, through the accompanying social dynamics, to wider socio-cultural contexts (Lee et al., 2022). The process of contextualisation may also refer to the development of situated understanding of robots that emerges from the real-world interactions (Šabanović, 2010) or the need to interpret research results within larger HRI multidisciplinary frameworks inclusive of SSH (Seibt et al., 2021). This offers multiple new topics and lines of inquiry, e.g., into socially-situated HRI (Chang and Šabanović, 2015) or feminist perspectives on the embodied and gendered user experience in HRI (Winkle et al., 2023a). From this perspective, contexts and situations can be understood not as “static containers for ideas, thoughts, and interactions” [(Linell and Valsiner, 2009): 16] but rather as resources that “dynamically change with the participants’ communicative and cognitive activities” (ibid.). The question is how to investigate these subjects in a way it helps advance not only the field of HRI but also the SSH-driven critique of the contemporary everyday life.

### 4.4 Sociality

Given the broad cross-disciplinary consensus on that humans are social beings (Enfield et al., 2006; Goodwin, 2018; Lee et al., 2022), human everyday life has often been seen as inherently and explicitly “social.” It has been argued that “the reality of the everyday life-world is a social reality” [(Pedersen, 2016): xxx], where human sociality is not just complex but also under many aspects “special” (Enfield et al., 2006). In other words, “the social world is nothing other than an interpersonal field, an inter-human space. . . this embeddedness of human beings in the relationships with other human beings occurs nowhere else but in our everyday experiences” [(Dumouchel and Damiano, 2017): 30]. From this perspective, human life-worlds are neither private nor public but shared, i.e., built upon intersubjectivity and common experiences [(Pedersen, 2016): 68]. Lived experiences in particular may contain specific forms of sociality, i.e., “various forms of the experience of others” [(Pedersen, 2016): 27]. Of course, the concept and experience of sociality and human embeddedness in larger social structures continues to change, particularly in the present age of radical individualisation (Sztompka and Burawoy, 2011). Also, the very understanding of what makes a given domain or activity private or public is highly culture-specific, and the notion of privacy is being constantly redefined as the use of different digital technologies increases. In any case, the key focus of the SSH analyses of everyday life is on “the social,” and it is also the crucial aspect of HRI research. It has been argued that investigations of human interactions with social robots, and hence, social interactions, is what constitutes the core of HRI and makes it unique as a discipline (Bartneck et al., 2020). This includes studies of what makes human-robot interactions a “social experience” and how to deliver it (Burch, 1990; Husserl, 2002), and occasionally the studies that explicitly address people’s experiences of living with social robots in home settings (Heidegger, 1967; Sarte, 1984; Huang and Huang, 2021). However, assessing people’s lived experiences regarding social robots is a difficult task with multiple variables and perspectives involved (de Beuvoir, 1953) that is yet to be fully addressed in HRI. The challenge is not only in developing suitable theoretical and methodological frameworks but also in recognising the importance of people’s lived experiences as such (rather than, e.g.,. prioritise designers’ and developers’ technical expert knowledge (Miller et al., 2021)) and systematically addressing such experiences in their whole richness and complexity within wider socio-cultural contexts (Prus, 1996; Merleau-Ponty, 2012).

## 5 Towards HRI of everyday life

Given its scope and diversity, developing a conceptual framework for studying everyday life in HRI is not an easy task and it needs to be undertaken carefully. For example, the ELCA approach explicitly refrains from pointing to what to focus on when investigating human-robot interactions in the everyday (Weiss and Hannibal, 2018). At the same time, if it is true that social robots “sooner or later” will become a part of our daily life, incorporating everyday life into HRI is also inevitable. Far from claiming that this a complete or even approximate framework, we discuss here the key dimensions that we find essential for further conceptualisation of everyday life in HRI as a distinct research theme. While building upon the existing SSH theories of the everyday, in this section we aim to envision those aspects or elements of everyday life that due to the presence of social robots will become significantly altered, new or “extraordinary.” It is also social robots with their specific applications, capabilities and limitations that will ultimately delineate boundaries for the otherwise infinite the human everyday we aim to address. Given a very limited presence of the actual social robots in our lives to date, and their significant novelty, research into human-robot daily life will require moving away from studying everyday life as the domain that is well-known and self-evident, towards ‘stepping into the unknown’ (just as any time when addressing “questions of change, futures and anticipated but as yet not experienced alterities” [(Pink et al., 2020): 135]). From this perspective, the HRI of everyday life focuses not on the everyday as we know it but as we make it.

### 5.1 Multi-sitedness

As discussed above, everyday life has typically been viewed in HRI as synonymous with a domestic life. It important to note however, that while home as a space and concept often holds a particular significance in people’s lives, our everyday life is multi-sited (Bakardjieva, 2005). This is particularly important in a situation where a distinction between the private and public is increasingly blurred, and the very notion of a household or what we consider a family and couple changes (Beck, 2001). Also, in the contemporary society, daily life has been increasingly taking place in virtual or otherwise technologicaly-mediated spaces, including ‘no-places’ such as the internet. While multi-sited approaches are well-known in SSH, particularly in ethnographic research (Hasse, 2019; Pink et al., 2020), choosing specific sites for analysis is far from being a trivial task, and should always involve participation of the persons whose lives we actually study. Perhaps a useful analogy in this context that comes from the SSH research is that of ‘horizons’. In general, the term ‘horizons’ refers to frameworks or temporal and spatial perspectives within which people construct the meaning and value of their actions in the world [(Gardiner, 2000): 21; (Taylor, 1989): 27], or the “inner and outer horizons” of all everyday experiences [(Schutz and Luckmann, 1973): 167] (or a “double horizon” of both external and bodily space described by Merleau-Ponty, as discussed in (Katz and Csordas, 2003)). On the one hand, using a horizon analogy includes looking at everyday life in terms of one’s immediate experiences and limits within particular “life-worlds”; on the other hand, it also points to the potential and possibilities for human action beyond the life horizon (Schutz and Luckmann, 1973; Pickering, 2004; Bakardjieva, 2005). The use and presence of social robots may potentially challenge and redefine the existing inner and outer horizons in people’s daily lives and link a number of dimensions our lives are made of into entirely new configurations. Also, we propose to have as a main frame of reference not a “site” understood as a specific geographical location but rather a “living space” of a person involved. Since human living spaces are inherently symbolic spaces (Seibt et al., 2021), they refer not only to spaces people and robots (will potentially) literally cohabit, where robots bring new meanings and values into such environments (Šabanović, 2010) but also social, moral, topical and other types of individual and common spaces, whether already existing or possible in future (Taylor, 1989; Taylor, 2002), that together constitute what a person experiences as ‘life’.

### 5.2 Human subjects

With an increasing importance of the studies “in-the-wild,” HRI research often involves human subjects. The term “human subjects” can be seen as synonymous with that of “participants” in this context (the two have often been used interchangeably, including in sociology (Giddens and Griffiths, 2006) or in different research ethics guidelines (Winkle et al., 2023a)) as it refers to the “real life people,” or “living individuals” that are directly involved in the studies (Bruckman, 2002). Given the focus on the people’s everyday experiences, we propose to address the study participants in HRI as ‘subjects’ in a wider sense, particularly in line with the classical sociological conception of the subject that to a large extent refers to the “person” (Black, 2000). Interestingly, in HRI community too there have also been discussions about how to conceptualise what is meant with ‘human subject’ in relation to their role (e.g., (Onnasch and Roesler, 2021; Lee et al., 2022)) and in broader terms of participation practices in HRI research (Winkle et al., 2023b). Without neglecting the intersubjectivity aspects, different sociological perspective follow the assumption that human beings are “acting and experiencing individuals” [(Overgaard et al., 2009): 101] who play an active role in shaping their own existence and giving meanings to the experiences such an existence involves. The emphasis on human subjects also allows contextualising the subject within the concrete historical reality a given person is part of [(Wieviorka and Tomasi, 2001): 82], and concrete biographic contexts. On the one hand, the key characteristic of the subject is its capacity to, at least in principle, assert personal liberty and ability to choose, while combining the universal with the particular; on the other hand, the notion of the subject points to the inherently social and intersubjective character of human condition, since “there can be no personal subject without the recognition of the subject in the Other” [(Wieviorka and Tomasi, 2001): 83]. From an analytical point of view, the focus on the human subjects allows a number of perspectives, e.g., a focus on the subject’s activity and individual practices located within larger institutional and collective frameworks, or the study of the corporeal subject and the body as its integral part [(Wieviorka and Tomasi, 2001): 83]. It also emphasises the need to look at the actual lived experiences and meanings they have for the people and communities involved in daily life, including life with social robots. Finally, the emphasis on human subjects is well-suited for the analysis of everyday life understood in terms of ‘life-worlds’.

### 5.3 Community focus

In order to advance our understanding of human daily life with social robots we emphasise the need to conduct HRI studies that focus on a “community.” This is because human existence in the real-world is of course always a social existence to a varying degree. Thus, in order to be accurate, HRI studies “in-the-wild” should involve more than robot interactions with single end-users to start with. We view the notion of community as more useful as, e.g., groups in this context as it allows explore unique characteristics and experiences social robots offer in their capacity for human-like engagement with people. Also, bringing focus to communities is part of the efforts to conceptualise the everyday as a reality involving concrete persons and circumstances, as opposed to “generalizable humans” (Lee et al., 2022), the approach that dominates HRI research. From an analytical point of view, community can be understood as a social unit, a process, or a way of life. The notion and experience of community inevitably changes as the human condition does (it is also one of those concepts that have long been declared dead by sociologists and yet it continues to return) (Day, 2006). For the purposes of this work, it important to note that community can be conceived as “an active process through which individuals and groups strive to realise their potential” [(Day, 2006): 21]. Also, community typically involves a sense of belonging to place and having one’s identity “wrapped up” within one’s community. This is in a situation where community is firmly embedded in the daily life of social actors involved, as it plays an important role in organising people’s day to day living (Day, 2006). Across different perspectives, the concept of community has often been associated with a notion of ‘a good life’ or otherwise positive experience (Taylor, 1989; Bauman, 2000; Day, 2006) (the approach sometimes criticised for being overly romantic when discussing traditional communities and their disappearance [(Day, 2006): 32] (Bauman, 2000)). Some works that have explicitly used such a notion in relation to social robots have also argued that the concept of a good life in this context should be understood as “a rich social life” [(Brand et al., 2023): 166]. Last but not least, communities can be seen as social and symbolic constructs that are “constituted by processes occurring close to the experience of everyday life” [(Day, 2006): 159]. From this perspective, it is people’s feelings and experiences that are the key defining characteristics of the community. The core of the communities are the processes of differentiation and identification, which includes identifying or imagining those who do and do not belong to the community based on a given criterion of similarity (Day, 2006).

On the one hand, community can be viewed as a taken-for-granted reality, and a stable setting for everyday social relationships; on the other hand, community can also be actively pursued and used as a framework for imagining and striving for a better life [(Day, 2006): 25]. Particularly in the contemporary times, definition and participation in a community becomes increasingly a matter of an individual choice and a conscious strategy rather than structural or institutional factors (Day, 2006). Therefore, we propose to conduct HRI studies that explore new elements that bring people together and serve as a common identifying criterion when dealing with social robots in daily life. More specifically, in a situation of an ever-increasing division, plurality and heterogeneity of the contemporary communities and societies as a whole, one could be asking if social robots can play a potentially unifying vs. diversifying role, or whether the realm of the everyday really is where people become “truly *human* persons” [(Gardiner, 2000): 2] [italics original]. This is because a largely unique feature of everyday life with social robots may be the role such robots will have in bringing our attention to otherwise taken-for-granted human qualities, and potentially redefining our understanding of what it means to be a human subject on both an individual and collective level. In other words, as part of the HRI research agenda, we propose to investigate whether a sense of humanness, as defined and experienced when sharing a daily life with social robots, can constitute an new basis for a “human togetherness” (Schloßberger, 2016) and provide a holistic perspective in the otherwise fragmented and individualised contemporary communal life.

### 5.4 Everyday in making

Both SSH and HRI research are highly ambitious in their attempts to address and analyse the overarching realms of “everyday life” and “real-world” respectively. In practice however, it is possible to address only fragments of these complex and dynamic realties, and construct selective representations of the phenomena we study. The question is, e.g., not only how to increase and facilitate longitudinal studies in HRI (De Graaf et al., 2017; Ostrowski et al., 2022) [in a situation where a long-term fieldwork becomes generally less and less feasible as much for researchers as the study participants involved (Pink et al., 2020)] but also how to conceptualise and empirically investigate the everyday in a way we consider sufficiently accurate and explanatory. The very role of study participants also changes, as it has been increasingly accepted that research should be conducted “with” rather than “about” people (Pink et al., 2020) [the idea underlying participatory approaches in HRI (Šabanović, 2010)], with people actively contributing to the construction of research findings that concern them. Even when reducing the scope to studying people’s lived experiences that involve social robots, one faces multiple social, practical and technical constraints and choices that often lead to only a quick and narrow glimpse into the human everyday life. That we can only obtain approximations of the phenomena we study is of course not unique to the field of SSH or HRI (Newell, 1982; Niiniluoto et al., 1986). In the case of HRI research, the challenge is even greater as to a large extent it involves investigating and envisioning “the future” rather than the actual everyday with social robots as it is now. The condition of not knowing that underlies any investigations of the future implies that research becomes a matter of “making” rather following a predefined research design (Pink et al., 2020). Therefore, we propose to conceptualise everyday life in HRI not as a domain that is largely familiar and taken-for-granted, and where social robots are supposed to ‘fit in’ to a varying degree, but rather as a territory for research and social experimentation like never before. Also, research into human-robot futures will need to consider all the challenges, risks and ethical dilemma that potentially come with it (the issues that the HRI community has to some extent already been reflecting on, particularly with regards to good scientific practices and search for disciplinary guidelines (Rosén et al., 2021)). By fully embracing the need to “step into the unknown” (Pink et al., 2020), both in terms of theories and methods used, HRI researchers would open the doors for exploiting the novelty or “extraordinary” elements that come with social robots, and embracing ‘the unexpected’ that is inherent to the HRI research “in-the-wild” (Lee et al., 2022).

## 6 The old/new everyday

In the HRI field, the human everyday social and physical world has often been considered ‘real’ or “natural.” In particular, conducting studies “in-the-wild” is viewed as useful in that they allow addressing “natural” human behaviours and interactions with robots (Lee et al., 2022) as well as the circumstances that are also defined as “natural” [(Bartneck et al., 2020): 144]. The investigation of “natural” human environments or interaction settings has often been explicitly linked with the use of robots in everyday life (Šabanović et al., 2014a; Rosenthal-von der Pütten et al., 2016). Many HRI studies address “human nature,” which involves the tendency to represent humans as “generalizable humans” (Lee et al., 2022). The very “natural interactions” between robots and humans have sometimes been seen as synonymous with “*normal interactions in everyday life*” [(Ferland et al., 2013): 118][italics original]. In its capacity to point to the “normal,” “everyday” and essentially “human,” the term “natural” (or the notion of a “real-world”) is often taken-for-granted in HRI (or in SSH when, e.g., referring to “real social life” [(Linell and Valsiner, 2009): 5]). It is important to note however, that any assumptions about what is “natural” or “normal” for people immediately pose a whole range of questions and ethical dilemma, particularly regarding inclusion criteria. The very notion and experience of humanness is of course by no means homogenous, and different related claims of its universality have been questioned (Tharoor, 1990; Peterson, 2001; Valentine, 2017). At the same time, current robotics technologies are expected to bring profound changes across different domains, the process that in turn is viewed as part of larger fundamental transformation taking place in our society which includes the emergence of “a new kind of everyday life” [(Beck, 2001): 262].

Therefore, we argue here that if we agree that everyday life is not a fixed feature of social life [(Gardiner, 2000): 10], the next important role for HRI research may include not only helping conceptualise and systematically study human daily life with social robots but also engaging in a critique of the contemporary everyday life. Among its different meanings (Anker et al., 2017), we use the term “critique” to denote a specific method and type of argument made in academia with the goal to bring a social and political transformation. In particular, we propose to develop a critical approach and mode of inquiry that would be grounded in the unique features, capabilities and empirical insights of the HRI research. From this perspective, a primary role for HRI research could include contributing to a “diagnose” of the current everyday, and ultimately contribute to our quest for “a good” (or “better”) life. Thus, in addition to studying human-robot interactions, one could be asking what are the big themes that HRI can shed light on that point to those aspects or elements of everyday life that need to be improved? For example, the use and presence of social robots has often been discussed in the context of a growing social isolation taking place in techno-capitalist societies (Šabanović, 2010; Ananto and Young, 2021). Another example of a critical HRI inquiry includes recent developments in the “Feminist HRI” (Winkle et al., 2021; Winkle et al., 2023a) or reflections on human adaptation (or a lack of thereof) to life with robots (Harrison and Johnson, 2023). What are the other aspects of not so much “human nature” as of the contemporary human condition that HRI and social robots could potentially unmask and transfigure? In the times of the increasing “homogenization of the concrete particularities of the everyday lifeworld” [(Gardiner, 2000): 13], but also “the confrontation with fundamental differences everywhere and the necessity to live (with) these contradictory certainties somehow” [(Beck and Tomasi, 2001): 189], what are the potential implications of exploiting the similarities vs. differences between “the human” and “the human-like” that come with social robots? As discussed above, familiarity in everyday life is usually graspable in the negative [(Schutz and Luckmann, 1973): 159], i.e., when familiar elements suddenly become unfamiliar to us, and before the “new” becomes “old.” Thus, if we agree that critical thinking is “part of the everyday experiences of individuals forced to negotiate between conflicting spheres of value in complex societies” [(Anker et al., 2017): 14], then we may see how a critique of everyday life can be pursued with the use and presence of social robots that often call into question our existing views of what is “normal” in daily life or what it means to be human. One could argue that as much as contemporary technologies challenge any fixed concept of humanness (Capurro et al., 2006; Miah, 2008), when confronted with highly human-like AI and robotic systems, people tend to unite and perceive themselves as universally “human.” Particularly when considering threats inherent to emergent technologies such as AI, the notion of “human” has often been used in affirmative terms, leading to the creation of a new type of solidarity among humans (“us”) as opposed to “them” (AI-enabled systems). From this perspective, everyday experiences with social robots may lead to new ways to identify as a human being, and new types of human communities. Of course, the implications of everyday life and human engagement with social robots can be not only beneficial but also problematic and should be addressed as such. For example, what contradictions or paradoxes may potentially arise in people’s everyday lives when shared with social robots? What would be the best course of action to deal with unexpected effects of robotisation?

It is important to note that since critique is typically characterised by a high degree of self-reflexivity, the matters that are particularly suitable for a critical analysis are those that appear to be self-evident: “Whatever is natural, taken for granted, essentialised or transparent become the critic’s target: such qualities are seen as not only theoretically inadequate (in failing to acknowledge the linguistic and cultural construction of reality), but also politically troubling (in “naturalizing” social phenomena and thereby rendering them immune to criticism and change)” [(Anker et al., 2017): 8]. We argue here that engaging with the critique in the HRI field would be instrumental not only in helping bring a desired social change but also critically examine its own underlying assumptions and normative beliefs. The need for a critical analysis will be even greater the moment social robots become ‘transparent’ or invisible in our everyday lives (if ever), just as some other technologies that disappear from human experience and focus (Verbeek, 2012). A unique potential of HRI research is in uncovering meanings and phenomena through empirical analysis rather than only a specific mode of reading or interpretation that critique typically relies upon.

As already mentioned, in principle critique is not only a specific research tool or theory but also a practice that requires taking an explicit ethico-political stance, and a commitment to bringing social and political change [(Gardiner, 2000): 9][(Anker et al., 2017): 13]. While it has been increasingly recognised that HRI research should be ethically- and socially-engaged (Arnold and Scheutz, 2017; Vallès-Peris et al., 2018; Lee et al., 2022), a discussion of a political role that HRI may play however (not just of the political issues social robotics technologies may cause), is yet to take place. In other words, what are the political motivations, goals and interests for HRI as a field and community, and how can it make a political difference, if any? In line with recent developments in the area of critical studies (Anker et al., 2017), critique can be seen not just as a form of opposition or negation but rather an active and purposeful response to a given problem. One could argue that given the enormous efforts and expectations placed in robotics technologies, along with the related cross-sectorial involvement of a variety of public and private actors, social robots and HRI can have a very real and powerful impact on the current political and social world. To what extent such an impact will actually materialise, and whether it will be positive, is yet to be seen. However, the potential of social robotics to change people’s everyday lives can certainly serve as both strong motivation and a fascinating challenge in the current HRI research.

## 7 Conclusion

In order to further advance HRI research, there can be no HRI studies of human daily life with robots without systematically addressing and conceptualising the key problems of the everyday as it unfolds in the “real-world.” At the same time, there can be no empirically-grounded SSH research on social robots without actually engaging with robotics technologies and related disciplines. This is why there is a need and great potential in combining HRI and SSH perspectives and using such a combination as a basis for further developments of uniquely new approaches dedicated to the theme of everyday life and human lived experiences with social robots. The questions we have posed here can be seen as part of calls for pursuing HRI research that is socially-engaged (Lee et al., 2022) or a critical examination of tacit values and underlying socio-cultural factors that to a large extent shape the design and use of robots (Šabanović et al., 2014b; Šabanović, 2014; Čaić et al., 2018). In particular, the point that we have hoped to emphasise is that HRI research has the potential to significantly expand its range of theoretical and methodological perspectives, role and ultimately political commitments, to help conceptualise, study and actively create the old/new human everyday with social robots grounded in what we all consider a communal “good life”.

## Data Availability

The original contributions presented in the study are included in the article/supplementary materials, further inquiries can be directed to the corresponding authors.

## Footnotes

1 What exactly counts as a “real world” and for whom is a whole different question. The imperative of “going to the real-world” is of course not unique to HRI as it has been underlying many other disciplines, particularly sociology.
